# Lotus Effect and Friction: Does Nonsticky Mean Slippery?

**DOI:** 10.3390/biomimetics5020028

**Published:** 2020-06-12

**Authors:** Md Syam Hasan, Michael Nosonovsky

**Affiliations:** Mechanical Engineering Department, University of Wisconsin-Milwaukee, 3200 N Cramer St, Milwaukee, WI 53211, USA; mdsyam@uwm.edu

**Keywords:** wetting, friction, adhesion, biomimetic surfaces

## Abstract

Lotus-effect-based superhydrophobicity is one of the most celebrated applications of biomimetics in materials science. Due to a combination of controlled surface roughness (surface patterns) and low-surface energy coatings, superhydrophobic surfaces repel water and, to some extent, other liquids. However, many applications require surfaces which are water-repellent but provide high friction. An example would be highway or runway pavements, which should support high wheel–pavement traction. Despite a common perception that making a surface non-wet also makes it slippery, the correlation between non-wetting and low friction is not always direct. This is because friction and wetting involve many mechanisms and because adhesion cannot be characterized by a single factor. We review relevant adhesion mechanisms and parameters (the interfacial energy, contact angle, contact angle hysteresis, and specific fracture energy) and discuss the complex interrelation between friction and wetting, which is crucial for the design of biomimetic functional surfaces.

## 1. Introduction

Biomimetics is mimicking living nature for engineering applications. The term “biomimetics” was suggested by Otto Schmitt in the 1950s. A similar concept was developed also by Jack E. Steele in 1958 under the name “bionics.” Both concepts were popularized during the next decades; however, today the word “bionics” is common mostly in popular culture, while “biomimetics” is used in scientific and engineering literature. More rigorous definitions (such as the International Organization for Standardization Standard 18458 of 2015) distinguish between *biomimetics* (“an interdisciplinary cooperation of biology and technology or other fields of innovation with the goal of solving practical problems through the function analysis of biological systems, their abstraction into models, and the transfer into and application of these models to the solution”), *bionics* (“a technical discipline that seeks to replicate, increase, or replace biological functions by their electronic and/or mechanical equivalents”), *biomimicry* or *biomimetism* (“philosophy and interdisciplinary design approaches taking nature as a model to meet the challenges of sustainable development”), and *bioinspiration* (“a creative approach based on the observation of biological systems”) [1].

Biomimetic approaches are currently quite common in a wide range of fields including materials science, artificial intelligence, robotics, biomedical applications, and so on. Particularly important applications are found in materials science, and they include self-healing, self-lubricating, and self-cleaning materials and surfaces [2].

The most common example of a successful use of biomimetics in surface science is the lotus effect for water-repellency and self-cleaning. Among natural leaves, the lotus (*Nelumbo nucifera*) leaf exhibits extreme water repellence and hydrophobic behavior. When a water droplet is placed on a lotus leaf surface, the adhesion between the water and dust particles is greater than the adhesion between the dust and the leaf surface. As a result, the water droplet picks up the dust particles and rolls off the leaf surface immediately. The lotus (पुण्डरीक (*Puṇḍarīka*) or पद्म (*Padma*) in Sanskrit, পুণ্ডরীক in Bengali, and 蓮華 (Liánhuá) in Chinese) became a symbol of purity in many Asian cultures, as reflected in Hindu and Buddhist sacred texts such as *Bhagavat Gita* 5:10 (“Having abandoned attachment he acts unaffected by Evil, as Lotus leaf is not wetted”) or the *Lotus Sutra* 14:46 (“They have learned the bodhisattva way and are untainted by worldly things, just as the lotus flower in the water”); the lotus (ﺳﺪﺮ) is also mentioned as a symbol of purity in the *Quran* (*Al-Waqi‘ah* 56:28) and the *Hadith* (The Book of Purification 1:189) [1,3,4]. There are more than 70 words for lotus in Sanskrit [4].

The superhydrophobic and self-cleaning mechanism of the lotus leaf is due to a special hierarchical roughness profile of its surface combined with wax coating, and it is called the “lotus effect” [5]. Quantitively, superhydrophobic surfaces are characterized by high values of the apparent water contact angle (CA > 150°) and low values of contact angle hysteresis (CAH < 10°). Recent advancements in micro-/nanotechnology have made it possible to design biomimetic surfaces with desired properties like non-adhesiveness, water repellence or superhydrophobicity, icephobicity, self-cleaning capacity, and so on. For example, biomimetic superhydrophobic surfaces are synthesized by using low surface energy coatings or by introducing micro-/nano-level roughness on the surfaces [6].

Superhydrophobic surfaces are characterized by low adhesion, which is due to the low surface free energy. Therefore, materials having the low surface free energy are used to manufacture superhydrophobic, non-adhesive biomimetic surfaces [7]. A variety of nonsticky, low-adhesion fluorine polymers, polytetrafluoroethylene (PTFE; popularly known under its commercial name “Teflon”), are used in different applications. For a smooth PTFE surface, the water CA varies between 109° to 114° and the surface free energy is low. The surface free energy of a PTFE surface can have a minimum value of $22{\;\mathrm{mJ}\;m}^{-2}$ [8,9].

The concept of surface free energy can be used to analyze the wetting phenomena. The surface free energy is often considered equivalent to the surface tension though they are not identical concepts. Surface tension (measured in N/m) is commonly defined as the force acting along the interface but perpendicular to the three-phase contact line. On the other hand, the surface free energy is measured in ${J\;m}^{-2}$ and it is equal to the energy required to form a surface with a unit area. At the surface, the atoms and molecules have fewer bonds with neighboring atoms and molecules compared to the bulk. As a result, they possess higher energy than the atoms and molecules in the bulk. This excess energy of the surface atoms and molecules contributes to the surface free energy. The CA measurement is one of the most popular methods of determining the surface free energy. In this method, the CA of the surface is measured using different liquids (e.g., water and diiodomethane). Using the CA data and knowing the surface tension forces of the liquids, the surface free energy can be calculated.

When a liquid droplet is placed on a solid surface, the solid and liquid surfaces reach an equilibrium state, which corresponds to the minimum energy state of the three phases. In an ideal situation for a smooth and homogeneous surface, the equilibrium value of the most stable contact angle (CA), $\theta_{0}$, is expressed by the Young equation:

(1) $$cos\;\theta_{0}=\frac{\gamma_{sv}-\gamma_{sl}}{\gamma_{lv}}$$

where $\gamma_{sv},\;\gamma_{lv},\;\mathrm{and}\;\gamma_{sl}$ are the surface tension forces (or interfacial energy) of the solid–vapor, the liquid–vapor, and the solid–liquid interfaces, respectively, acting on the contact line.

The formation of adhesive bonds of solids in contact is influenced by the surface free energy. As a result, the surface free energy influences the frictional behavior of sliding surfaces. This is because the two main factors contributing to friction are the adhesion between contacting surfaces and surface roughness. There are differences in the findings of different researchers about the relation between the surface free energy and friction. Several researchers suggested that there is a linear relationship between the surface free energy and the coefficient of friction (COF) [10,11]. A large surface free energy leads to high adhesion in a solid–solid contact, which eventually results in an increase in the COF. Other researchers suggested that there is no general relationship between friction and surface free energy [12]. The relationship between the surface free energy and friction is influenced by a lot of other factors, which makes it complex.

Biomimetic superhydrophobic surfaces can be effective solutions in combating corrosion and water-induced damages. However, there is a common perception that, for “nonsticky”, water-repellent, hydrophobic/superhydrophobic surfaces, the COF of solid–solid contact is reduced [13]. One can argue that the dry solid–solid friction depends on macro- and microscale roughness of the surface more than on the chemical nature of the surface [14]. Therefore, the question arises whether it is practical to use biomimetic, hydrophobic/superhydrophobic surfaces in applications that require water repellence and high friction at the same time (e.g., pavements, highways, and runways). Therefore, finding the correlation between the wetting and frictional properties of biomimetic hydrophobic/superhydrophobic becomes important.

**Figure 1:**
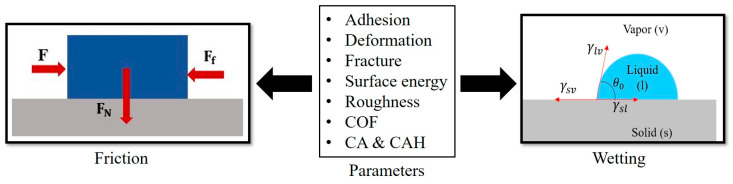
Parameters interrelating friction and wetting.

In this study, we review relevant adhesion mechanisms and parameters and strive to find the complex interrelation between friction and wetting, investigating the effect of the surface free energy and other surface parameters on both frictional and wetting properties (Figure 1).

## 2. Role of Adhesion in Friction

The two major factors which contribute to friction are adhesion and surface roughness. Adhesion is closely related to the surface energy and wetting; however, the role of adhesion in friction is quite complex.

### 2.1. Adhesion vs. Deformation in Frictional Mechanisms

In this section, we will discuss the phenomenon of friction and the role of adhesion and deformation in frictional mechanisms. Friction is an important topic of physics and engineering with a great practical significance [15,16,17]. Friction is the resistance to motion, which is experienced in relative motion of solid surfaces, liquid layers, or material elements in contact. The resistive tangential force is the friction force. Dry friction, as the name implies, describes the friction between two solid surfaces.

The quantitative properties of friction between solid surfaces are generally subject to Amontions–Coulomb’s laws of friction (sometimes called “Coulomb’s laws”). The first law states that the friction force between the surface of two bodies is directly proportional to the normal load with which the two bodies are pressed together. The proportionality constant is the COF. The COF between two sliding surfaces is defined as the ratio of the frictional force ($F_{f}$) between them and the normal force, N (the force pressing them together).

(2) $$COF=\frac{F_{f}}{N}$$

According to the second law, the friction force and the COF do not depend on the apparent (or nominal) area of contact between the contacting bodies. This is because the contact occurs only on the tops of the asperities, and the real area of contact is only a small fraction of the apparent area of contact, at least for traditional engineering materials such as metals (however, for elastomers and soft materials, the nominal and real areas of contact can be close to each other).

The third law governs the kinetic friction, and it states that the friction force is independent of the sliding velocity. Amontons–Coulomb’s laws of friction are just empirical approximations and are not theoretically justified propositions. In many cases, especially at the micro- and nanoscales, Coulomb’s laws of friction are not valid. In particular, the friction force and the COF are not always independent of the apparent area of contact [18]. Several experimental results have also established that the COF is dependent on the size (macroscale vs. micro-/nanoscales), load, and sliding velocity [19,20].

**Figure 2:**
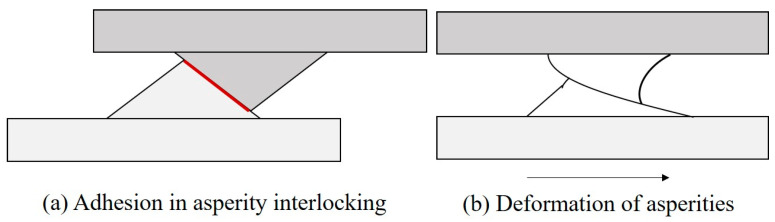
(**a**) Adhesion between interlocked asperities and (**b**) deformation of asperities in the shear direction.

The widely accepted theory of friction mechanisms was proposed by Bowen and Tabor [21]. They proposed that, for sliding contacts, friction mechanisms have two components: interfacial adhesion between asperities and asperity deformation (ploughing) at the real areas of contact between the surfaces (known as asperity contacts). The adhesion and deformation mechanisms are shown in Figure 2. There is negligible interaction between the adhesion component ($F_{a}$) and the deformation component ($F_{d}$) of friction, and the total friction force (*F*) can be presented as the sum of these two friction components.

(3) $$F=F_{a}+F_{d}$$

Adhesion occurs when dissimilar particles or solid surfaces are brought into contact under a certain normal loading condition or a combination of normal and shear loads. The molecular forces between the surfaces cause adhesion between them. The interaction force between the solids due to adhesion can be caused by the covalent, ionic, metallic, or van der Walls bonds. When two surfaces are placed in contact under load, the tip of the asperities of the two mating surfaces form the real area of contact. The physical and chemical interactions between the asperities result in adhesion at the interface [22]. The adhesion component $F_{a}$ of the friction force is proportional to the real area of contact ($A_{r}$) and to the shear strength ($\tau_{a}$) of the material. The friction force for a dry contact due to adhesion is defined as $F_{a}=A_{r}\tau_{a}$. In humid conditions or in the presence of lubricants, menisci or adhesive bridges may be formed at the interface, which has a significant influence on the overall friction force. Bhushan and Nosonovsky found that, during the sliding of two solid surfaces, the adhesion can be significantly increased in the presence of a liquid film of the condensate or a preexisting film of the liquid between them [23].

During sliding of two surfaces, the deformation component of friction is caused by both microscopic and macroscopic deformations. At the microscopic level, the displacements of the interlocked surfaces occur through the plastic deformation of the asperities. Asperities of the harder material plow grooves in the softer material.

Surface roughness and relative hardness of the two surfaces greatly influence the deformation component of friction. Reducing surface roughness reduces $F_{d}$. To maintain motion in the deformation process, the lateral force equal or exceeding $F_{d}$ is required. For perfectly smooth surfaces, no groove is produced through the deformation of the contacting bodies in sliding. In case of plowing, the shear strength of the material is proportional to the average value of the surface slope [24]. During plowing, wear particles of various sizes are generated. Wear particles along with contaminant particles trapped between the sliding surfaces form a so-called “third body”. The contacts take place on high asperities and particles [25]. The presence of the “third body” in plowing significantly increases the COF [26].

Friction is a complex phenomenon, with several mechanisms contributing to it. Adhesion and deformation have the most significant contributions to the overall friction force.

### 2.2. Friction of Metals, Polymers, and Rubber

General tendencies in friction mechanisms were discussed in the preceding section. However, particular sliding friction mechanisms can be different for different materials. For example, for friction of metals, the deformation component is dominant, while for polymers, the adhesion component is more significant. In this section, we will discuss the frictional mechanism and behavior of different materials including metals, metal alloys, polymers, and elastomers such as rubber.

For sliding friction, the deformation component of friction is dominant over the adhesion component. The deformation component incorporates the force required for plowing and grooving of surfaces. Irreversible plastic deformation is the dominant energy dissipation mechanism in a metal–metal contact, while only little energy is lost in mostly reversible elastic deformation [27]. The presence of any contaminant in the contact region can significantly reduce contact and friction between the two sliding surfaces. In the presence of air, the formation of an oxide film can separate the mating surfaces and can reduce the adhesion and friction [21]. Noble metals (e.g., silver, gold, and platinum) resist oxidation and exhibit high sliding COFs. Soft and ductile metals (e.g., lead and tin) exhibit high sliding COFs due to a large contact area and a small elastic recovery. For an alloy, generally, the COF is lower than that of its components [28].

The frictional behavior of polymers is significantly different from metals and alloys. The contact of a polymer with another polymer or metal is predominantly elastic. During sliding, the deformation component of friction results from the resistance of the material of one surface to be plowed by the asperities of the other. In the plowing process, asperities of polymer surfaces undergo plastic, elastic, and viscoelastic deformations. Adhesive bonds between the sliding polymer surfaces result in the adhesion component of friction. In friction of polymers, the adhesion component surpasses the deformation component [29]. In characterizing the friction of polymers, the formation and interaction of the polymeric transfer layers play an important role. Menezes et al. found that the transfer layer formation in polymer–steel contacts is controlled by the surface texture [30]. The formation of a polymeric transfer layer causes the reduction of the COF and the wear rate [31]. On the other hand, an incomplete polymeric transfer layer due to the lack of wear debris causes an increase in the COF [32].

Rubber is a polymeric material (i.e., elastomer) with significant importance. The frictional behavior of rubber is unique. An extremely low elastic modulus accompanied by high internal friction of rubber is the main reason behind this. Synthetic and natural rubbers are used in modern pneumatic tires. As a result, rubber friction on different hard substrates is a topic of tremendous importance. Like other polymers, rubber friction is attributed to adhesion and deformation components. In rubber–pavement friction, pavement asperities cause pulsating deformations of the rubber. It results in viscoelastic energy dissipation which contributes to the deformation component of friction. The adhesion component is derived from the adhesive forces between rubber and the substrate. For rubber friction, the adhesion component of friction is dominant over the deformation component.

Notable progress has been made in modeling the friction behavior of rubber and other elastomers [33,34,35,36]. It was found that the COF of the rubber–road interaction is load-dependent. Also, the thread deformation causes viscoelastic energy dissipation and an increase of the COF. The rubber–road COF is not always a constant under varying tire loads. Smith and Uddin proposed a theoretical model of tire–pavement friction [37]. They suggested that, when the rubber self-adhesively envelops some of the microroughness of the contacted pavement, surface deformation hysteresis occurs. Due to the surface deformation hysteresis, a fourth rubber force is observed that is independent of tire loading. Persson studied rubber friction behavior for the tire–pavement contact and presented a theory of the hysteresis-based rubber friction [38]. He also suggested that, for a zero or low sliding velocity, only the largest asperities of the roughness profile of the road touch the rubber surface. In each contact region, the local pressure squeezes the rubber into many of the smaller-sized cavities that contribute to the rubber–road COF.

Depending upon the surface roughness and mechanical properties, different materials have varying adhesion and deformation characteristics. The contribution of these two components characterizes friction during a contact between the surfaces of different materials.

### 2.3. Measures of Friction: The Coefficient of Friction, Surface Free Energy, Surface Roughness, and Fractional Toughness

In the preceding sections, different mechanisms of friction and frictional characteristics of different materials have been discussed qualitatively. In this section, we will discuss different parameters for quantifying friction.

#### 2.3.1. COF as a Measure of Friction

The COF is the traditional method of quantifying friction between two interacting surfaces. Though sometimes the COF is convenient to use, it is not a sufficient parameter to quantify friction. In many situations, the COF cannot be used as a reliable parameter to measure friction.

Amontons–Coulomb’s law predicted the COF as a linear proportionality constant. However, the linear dependency of the friction force on the normal load is only valid for certain traditional materials (e.g., metals) under certain loading conditions. McFarlane and Tabor found that the COF is dependent on load [39]. For some composite materials, the COF shows entirely nonlinear behavior. The COF value for a similar pair of materials in sliding contact varies significantly. Engineering handbooks and tables warn the users and ask to take precautions in using the documented COF data because the approximate reference data often varies in a wide range. For example, the COF values for steel-on-steel sliding are reported between 0.09 to 0.6 in different references [40,41]. Since the COF is not a material constant, no reference value of the COF for a given material combination can be assigned [42]. If the value of the COF for a material combination is required, it can be found by experiment only.

**Figure 3:**
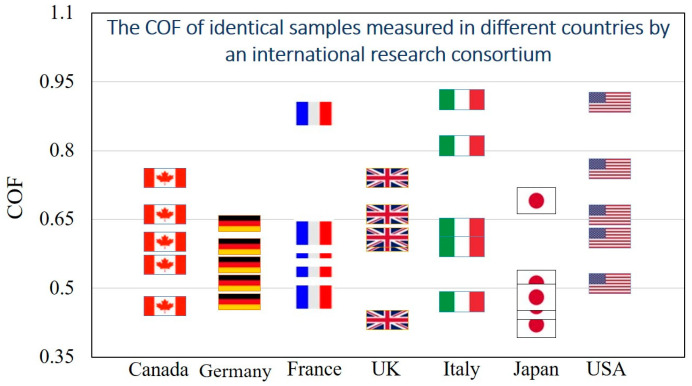
Coefficient of friction (COF) vs. country: Data on the COF of identical steel/aluminum-oxide sample pairs measured in different laboratories in different countries of the world. Each flag represents an experimental point from a laboratory.

The experimental values of the COF for the same material pair samples in sliding contact varies greatly. In a study by the Versailles Project on Advanced Materials and Standards (VAMAS) program in the 1990s, identical steel and aluminum oxide samples were sent to various laboratories across the world, in Canada, Germany, France, the UK, Italy, Japan, and the USA [43]. The samples had the same surface characteristics (i.e., roughness parameters), and identical laboratory environments were created to run the experiments for finding the COF for sliding contact. However, the measured COF values in different laboratories were found to be varying widely (between 0.4 to 0.9), resulting in what is known as “the dependency of the COF on country” (Figure 3). The large variation of the results, which apparently depended on poorly controlled and loosely defined factors during the experimental measurements, shows that the COF is not a very well-defined parameter.

The COF is scale-dependent, and it varies significantly at the macro- and nanoscales [18,20,44]. At the nanoscale, the surface-to-volume ratios are high and all surface effects, such as friction and adhesion, are significant and often dominant over the volumetric effects, such as inertia. There are different approaches to the scaling behavior of friction, which include the investigation of the scale effect on friction, scaling laws of friction, or the formulation of the nanoscale friction laws [18].

While Amontons–Coulomb’s laws are the foundation of tribology, these laws are not considered fundamental laws of nature but rather approximate empirical rules. Friction is perceived as a collective name for various unrelated effects of a different nature and of diverse mechanisms, such as adhesion, fracture, and deformation, lacking any internal unity or universality. For all of these reasons, although the COF is widely used, it is not a sufficient parameter to characterize friction properly. In the following sections, we will discuss other parameters that can characterize friction.

#### 2.3.2. Surface Free Energy as a Measure of Friction

The surface free energy of the sliding surfaces is closely related to their frictional behavior. Rabinowicz studied the influence of the surface free energy on friction and wear of sliding surfaces [45]. He proposed a mathematical model for finding the COF of sliding surfaces. He found that the COF for the sliding contact increases linearly with the increasing value of the surface energy. Nakao et al. studied the effect of the surface free energy on the COF of carbonaceous hard coatings. They found that the COF of various carbonaceous hard coatings decrease with the decreasing surface free energy of sliding surfaces. However, they found no proportional relationship between the COF and the surface free energy [11].

Kalin and Polajnar studied the effect of wetting and the surface free energy on friction for oil-lubricated friction [46]. They found that, for analyzing the effect of the surface free energy on the COF, it is convenient to use the spreading parameter (SP) instead of the CA:

(4) $$SP=\gamma_{sv}-\;\gamma_{lv}-\;\gamma_{sl}$$

where $\gamma_{sv}$,$\;\gamma_{lv}$, and $\gamma_{sl}$ are the surface tension forces (or interfacial surface energy) of the solid–vapor, the liquid–vapor, and the solid–liquid interfaces, respectively. They found that the smaller the value of SP, the smaller the COF for the sliding surface. A small value of the SP of a surface corresponds to a low surface free energy. Therefore, the smaller the surface free energy, the smaller the COF between the sliding surfaces. A low surface free energy causes a weak interaction between the surface and the lubricating oil. Due to the weak interaction, the slip is high and the COF between the sliding surfaces is low. On the other hand, there are other studies that claim that there is no direct relation between the COF and surface free energy. For example, Bäckström et al. studied the influence of the surface free energy on paper-to-paper friction [12]. They found no general relation between surface free energy and the COF.

The surface free energy is an important parameter to characterize frictional behavior. Generally, for two sliding surfaces, the larger the surface free energy, the larger the adhesion and, the larger the adhesion, the larger the friction.

#### 2.3.3. Surface Roughness and Friction

Dry sliding friction is greatly influenced by surface topography and surface roughness. There have been many theoretical and experimental studies to analyze surface topography and friction in terms of roughness parameters [47,48,49,50,51,52,53]. Various surface roughness parameters have been suggested to characterize the surface roughness.

Roughness parameters are generally classified into three categories: amplitude parameters, spacing parameters, and hybrid parameters. Amplitude parameters characterize the vertical deviation of the surface profile relative to a reference plane. The most common amplitude parameter is the average roughness, $R_{a}$, the arithmetic average of the absolute values of the profile height deviations from the mean line of the roughness profile. Mathematically, for a 2D roughness profile *z(x)*, $R_{a}$ is given by $R_{a}=\frac{1}{L}\int_{0}^{L}\left|z-m\right|dx$. Here, *L* is the sampling length of the profile and $m=\frac{1}{L}\int_{0}^{L}zdx$ is the mean value of *z*(*x*). Root mean square (RMS) roughness, $R_{q}$, is another amplitude parameter. The RMS is also known as the standard deviation, *σ*, and is given by $R_{q}=\sqrt{\left(\frac{1}{L}\int_{0}^{L}{\left(z-m\right)}^{2}dx\right)}$.

The skewness parameter characterizes the symmetry of the profile about the mean line. The mathematical formula of the skewness is given by $R_{sk}=\frac{1}{\sigma^{3}L}\int_{0}^{L}{\left(z-m\right)}^{3}dx$. The kurtosis is the roughness parameter that characterizes the sharpness of the profile, $R_{ku}=\frac{1}{\sigma^{4}L}\int_{0}^{L}{\left(z-m\right)}^{4}dx$. When surfaces with widely varying frictional characteristics have the same value of $R_{a}$, the kurtosis can be used to differentiate between them [50,51]. The maximum height of the profile, $R_{t}$, is another amplitude parameter defined as the absolute vertical distance between the highest profile peak, $R_{p},$ and the lowest profile valley, $R_{v}$, along the sampling length of the roughness profile, $R_{t}=R_{p}+R_{v}$. The maximum height is useful for studying the effect of surface roughness on sliding friction in presence of lubrication [49].

Besides these amplitude parameters, mean peak spacing, $S_{m}$; mean profile slope, $\Delta_{a}$; and core roughness depth, $R_{k}$, are important roughness parameters to characterize the COF [54]. These roughness parameters are related to the shape of the asperities of the surface profile. $S_{m}$ is defined as the mean spacing between peaks at the center line along the sampling length of the profile and given by the formula, $S_{m}=\frac{1}{n}\overset{n}{\underset{i=1}{\sum }}S_{i}\;$. Here, $S_{i}$ is the individual peak spacing and *n* is the total number of profile peaks. $\Delta_{a}$ is the average of all slopes ($\frac{\delta y_{i}}{\delta_{xi}}$) between each two successive points over the sampling length and is given by the formula $\Delta_{a}=\frac{1}{n-1}\overset{n-1}{\underset{i=1}{\sum }}\frac{\delta y_{i}}{\delta_{xi}}\;$. $R_{k}$ is obtained from the subtraction of the minimum and the maximum heights of the core surface profile.

A different approach is the characterization of a rough surface profile as a random signal. The relevant spacing parameter is the correlation length, which is the typical horizontal distance at which correlation in the levels at two arbitrary point become irrelevant.

Note that parameters traditionally used for the study of friction may be different from those used for the study of wetting. For biomimetic superhydrophobic surfaces, roughness is an important property. The traditional roughness parameters such as $R_{a}$, $R_{q}$, and $R_{ku}$ are not sufficient for the analysis of mechanisms involving superhydrophobicity [55]. Different roughness parameters are needed for this purpose. The CA, θ, of a rough solid surface having a roughness profile consisting of asperities and valleys was calculated by Wenzel as follows:

(5) $$cos\theta =R_{f}cos\theta_{0}$$

where $R_{f}$ is a roughness factor which is the ratio of the of the actual surface to the geometric surface. Nosonovsky and Bhushan showed that, for patterned surfaces, the nondimensional spacing factor, $S_{f}$, is an important parameter to characterize roughness [55]. For a patterned surface having circular flat-top pillars with diameter *D* and pitch *P*, $S_{f}=\frac{D}{P}$.

We discussed different traditional roughness parameters and their use in characterizing friction. Different surface roughness parameters have a strong correlation with the COF. For biomimetic superhydrophobic surfaces, apart from the traditional parameters, new roughness parameters are required.

#### 2.3.4. Fracture-Related Parameters

**Figure 4:**
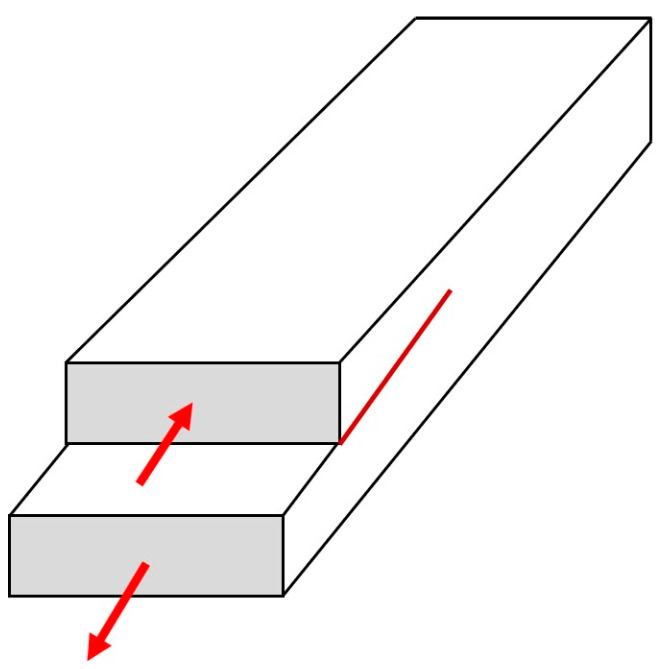
“Mode II” crack propagation for in-plane shear loading, red arrows showing applied forces.

A different approach to friction has been suggested by some physicists who emphasize the similarity between sliding and the shear mode crack (called in fracture mechanics “Mode II crack”) propagation [56]. The Mode II crack propagation for in-plane shear loading is shown in Figure 4. Svetlizky and Fineberg investigated the onset of frictional sliding between two polymethylmethacrylate (PMMA) blocks by measuring the real area of contact *A_r_* and strain fields near the interface between the blocks [57]. They found that the stress distribution at the transition from static to dynamic friction is in good quantitative agreement with the singular solutions for the motion of a rapid shear crack, as predicted by linear elastic fracture mechanics.

Ben David et al. found that nucleation locations are often regions where the shear-to-normal stress ratio, τ(x)/σ(x), is at the maximum [42]. They suggested that the effective fracture energy Γ reflects the local adhesive strength of the interface, as determined by σ(x), which is proportional to the real area of contact at every point. Consequently, Γ is not a material-dependent quantity as in the fracture of bulk materials but, instead, reflects the local strength of the interface. For a bulk material, it is often assumed to be twice the surface free energy of the material: Г = 2γ. For a frictional rapture, the effective value of Г is much smaller than γ because the real area of contact is only a small fraction of the apparent area of contact. On the other hand, τ(x) is proportional to the density of strain energy stored. The ratio τ(x)/σ(x) reflects the balance between the potential energy available before rupture in the vicinity of each point and the energy needed to rupture the interface. The locations where τ(x)/σ(x) is at maximum depend upon many random factors, such as the surface roughness, loading distortions, and imperfections. Consequently, it is difficult to attribute a value of the COF as a critical ratio of shear-to-normal load. The crack tip velocity is controlled by the balance of the specific fracture energy and the dynamic energy release rate, *G*.

Shlomai and Fineberg have studied experimentally the onset of frictional sliding at a bimaterial interface between polycarbonate (PC) sliding on polymethylmethacrylate (PMMA) with approximately 40% mismatch of the elastic wave velocities [58]. They observed a propagating slip-pulse, which could be interpreted as a manifestation of the Adams [59] instabilities, following a similar assessment by Ben-Zion [60] of the instabilities observed in the dynamic ruptures of earthquake faults, such as the San Andreas fault in California. These dynamic instabilities were predicted for the frictional sliding of two moderately dissimilar (in terms of their elastic properties) smooth elastic half-spaces with a nonzero constant COF between them. Later, the same phenomenon was also found for non-smooth (e.g., wavy) surfaces with zones of asperity contacts and separation [61]. The propagation of a train of frictional sliding pulses can result in an effective decrease of the apparent or observed COF in comparison with the local value of the COF [62].

The fracture mechanics-based approach (often used for the study of earthquakes [63]) uses quantitative parameters, alternative both to the COF employed by engineers and to free surface energy used in physical chemistry. These alternative parameters are the effective fracture energy and the dynamic energy release rate.

## 3. Adhesion, Surface Roughness, and Superhydrophobicity

In this section, the adhesion mechanisms in wetting will be discussed. We will analyze the contact angle hysteresis (CAH) as a measure of friction. Finally, the effects of the surface roughness and adhesion on wetting phenomena will be discussed.

### 3.1. Contact Angle vs. Contact Angle Hysteresis

The CA between a liquid droplet and a solid surface is used to quantify the wettability of the surface by the liquid. A hydrophilic surface is wet by water if a water droplet placed on it produces a CA less than 90°. On the other hand, on a hydrophobic surface, a water droplet produces a CA greater than 90°. A hydrophobic surface is characterized by a low surface energy. Due to its low surface energy, the contact area and adhesion between the solid surface and a water droplet are small. When the CA exceeds 150°, the surface is superhydrophobic [64]. A superhydrophobic surface has a very low surface energy. As a result, the contact area and adhesion between the solid surface and the liquid droplet are also very small.

**Figure 5:**
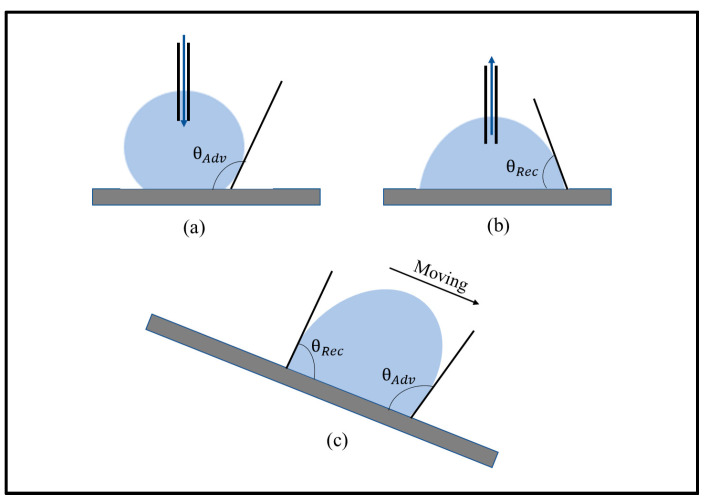
(**a**,**b**) Schematics of the sessile droplet method to measure contact angle hysteresis (CAH) and (**c**) a schematic of advancing and receding contact angles on the tilting plate.

The Young equation (Equation (1)) provides the observed (apparent) equilibrium value of the CA. The equilibrium value is obtained experimentally by placing accurately a sessile droplet on a solid surface so that it is in equilibrium. However, wetting scenarios may be much more complex. Sometimes the CA depends on whether liquid advances or recedes on the substrate. The advancing CA ($\theta_{Adv}$) corresponds to the maximum value of the CA on a solid substrate when a liquid droplet is placed on it, and the receding CA ($\theta_{Rec}$) corresponds to the minimum value. The sessile droplet method and the tilting plate method of measuring $\theta_{Adv}$ and $\theta_{Rec}$ are shown in Figure 5a–c.

The difference between the advancing and the receding contact angles is termed as the contact angle hysteresis (CAH). The CA and CAH give an indication of the adhesion between water and a solid surface [65]. Chemical and topographical heterogeneity (e.g., microroughness) of the surface contributes to CAH [66]. Contact line (CL) is the line where the liquid, solid, and vapor phases meet. Due to CAH, the CL is pinned in metastable positions. As a result, the sliding or rolling of the water droplet on an inclined surface is resisted. There are notable commonalities between solid–solid and solid–liquid friction. In a solid–solid contact, adhesion hysteresis (AH) is the difference between the energy required to separate two surfaces and the energy gained by bringing them in contact. Frictional shear stress is contributed by the AH in adhesive dry friction. In a solid–liquid contact, the CAH is also influenced by AH [24]. Due to the AH, the solid–liquid contact area changes. As a result, it affects the CA and CAH. For a liquid–solid contact, CAH can be a measure to characterize friction.

The effects of micro- and macroscale roughness on surface wetting properties are well established. The characterization and synthesis of superhydrophobic and hydrophilic surfaces have been studied extensively over the years [67,68,69,70,71]. Whether a surface is hydrophobic or hydrophilic depends upon the adhesion of water molecules to the solid surface. Usually, hydrophobic/superhydrophobic surfaces are characterized by a high CA and low CAH. A high CA value indicates a low liquid–solid adhesion. Also, a low CA hysteresis corresponds to a low liquid–solid adhesion. Unusually, a surface can have a large CA (hydrophobic) and can exhibit large CAH (strong adhesion to water) simultaneously [72]. In nature, this phenomenon is found in the petals of the red rose and is known as the “rose petal effect” [73,74]. Feng et al. studied the “rose petal effect” and found the existence of nanofolds on each micropapilla top [75]. A red rose petal has a close array of such micropapillae on its surface. They found that these hierarchical micro- and nanostructures provide enough roughness for superhydrophobicity in a rose petal. At the same time, they generate a high adhesion to water, which ensures that a water droplet does not roll off even when the petal is turned upside down.

To summarize, adhesion and roughness significantly influence the wetting properties. The CA and CAH are the common indicators of solid–liquid adhesion. Roughness contributes to pinning the triple line causing CAH. Also, CAH provides information about the solid–liquid friction.

### 3.2. Lotus-Effect-Based Superhydrophobic Materials

Roughness at the nano- and microlevels has significant effects on the CA and CAH, which is observed naturally in lotus leaves. The lotus effect and lotus-effect-based superhydrophobicity have been studied extensively in recent years [1,76,77,78,79,80]. In the study of two German botanists, Barthlott and Neinhuis, the unique dual scale micro-/nanostructures of the lotus leaves were revealed [81]. They found that the unique hierarchical micro-/nanostructures of a lotus leaf is due to a low surface energy epicuticular wax crystalloids and to a cuticular surface, which is highly rough at the micrometer scale. The combination of the dual scale roughness and low surface energy wax allows air to be trapped under the floating water drops that causes the “lotus effect” [82]. Consequently, small solid–liquid adhesion, a high CA, and a low CAH are observed.

There have been many efforts to synthesize lotus-effect-based superhydrophobic materials for different applications. For example, Kim et al. studied the process of synthesizing a surface with a hierarchical structure similar to the lotus leaf [83]. They were able to successfully mimic the superhydrophobic properties of the lotus leaf by dipping sandblasted porous alumina into polytetrafluoroethylene (PTFE) solution.

Liu and Li suggested a method of combining the lotus effect with the biomimetic shark skin effect. They used polydimethylsiloxane (PDMS) containing nano-silica as a substrate and treated it with heat. They were able to synthesize a surface having both the shark-skin surface morphology and the lotus leaf-like hierarchical micro-/nanostructures [84]. Yang et al. proposed the fabrication of a superhydrophobic coating mimicking the lotus leaf effect using strawberry-like Janus hemispherical particles [85]. The flexibility of the fabrication process allows the synthesis of unique coatings on substrates with varied composition and shape. Also, the coatings were durable enough to tolerate organic solvents and high-water flushing speeds. Haghdoost and Pitchumani adopted an electrodeposition technique for the fabrication of superhydrophobic surfaces [86]. Through a two-step electrodeposition process in a concentrated copper sulfate bath, they synthesized a copper deposit having multiscale surface textures leading to a high CA and low CAH.

Synthesizing omniphobic materials that repel water and oils is a greater challenge than synthesizing superhydrophobic materials. The challenge is due to a low surface energy and the nonpolar character of oil molecules. Wong et al. reported a novel approach of synthesizing biomimetic omniphobic surfaces which they named “slippery liquid-infused porous surfaces” (SLIPS) inspired by *Nepenthes* pitcher plants [87]. In the case of *Nepenthes* pitcher plants, impinging liquids are locked in on the surface. This intermediary liquid combined with microtextural roughness of the surface forms a slippery, inert, and continuous interface which provides omniphobic properties to pitcher plants. Mimicking this unique characteristic of pitcher plants, a continuous film of a lubricating oil is reported to be locked in place by a substrate having a micro-/nanoporous structure.

The lotus-effect-based superhydrophobicity is a popular type of biomimetic superhydrophobic surface, but the lotus leaves are not the only inspiration for synthesizing liquid repellent materials.

## 4. Friction in Fluid-Lubricated Contacts

**Figure 6:**
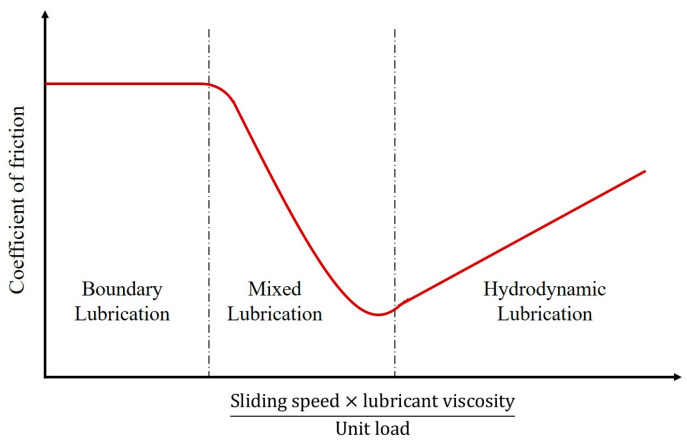
The Stribeck curve for fluid-lubricated contacts.

For the contact of fluid-lubricated surfaces, different lubrication regimes define the frictional behavior. The Stribeck curve for fluid-lubricated contacts is shown in Figure 6. The Stribeck curve presents the change in the COF of two fluid-lubricated surfaces against a dimensionless lubrication parameter, the Hersey number [88]. The dimensionless Hersey number is defined by the following equation:

(6) $$Hersey\;number=\frac{\eta V}{P}$$

where η is the dynamic viscosity of the fluid, *V* is the sliding velocity, and *P* is the unit normal load at the contact. From the Stribeck curve, it is seen that, for a fixed normal load and fluid viscosity, the COF between two fluid-lubricated surfaces depends on the sliding velocity.

Three distinct lubrication regimes are identified from the Stribeck curve. The first regime is known as the boundary lubrication. The boundary lubrication regime is characterized by a high COF, as surface asperities mainly support the load and the two surfaces have a direct contact. In the mixed lubrication regime, both surface asperities and the lubricant support the load. In the hydrodynamic lubrication regime, a negligible asperity contact is observed, the load is supported by the hydrodynamic pressure of the lubricant, and the COF is low.

## 5. Correlation between Friction and Wetting

Studying the relationship between friction and wetting is important for different theoretical and practical applications. However, due to the complexity involved to characterize wetting and friction, it is difficult to correlate them properly. There are only a few studies of an interrelation between friction and wetting available in literature. In this section, we will review some research works that intend to correlate friction and wetting.

Borruto and coworkers studied the effect of surface wettability on friction. In their tribological study, they used different combinations of hydrophilic (steel and pyrex glass) and hydrophobic materials (carbon fiber and PTFE) for sliding contact [89]. For the tribological characterization, a tribometer was used, and for the wetting characterization, CA measurements were done. The tribological tests were performed in three environments (dry, water lubrication, and oil lubrication). For hydrophilic–hydrophilic contacts, the COF was high in dry friction. In the presence of water, a very thin layer of water formed between the two wettable surfaces, which reduced the COF. With a lubricant, the COF was further reduced. For hydrophobic–hydrophobic contacts in the presence of water, the hydrophobic surfaces did not allow the formation of a water film between them. Therefore, the presence of water during tribological tests did not affect the COF while it was high in dry friction and very low with oil. For sliding surfaces with different wettability (hydrophobic–hydrophilic), in the presence of water, a continuous layer of water was formed between the two surfaces. As a result, the COF was reduced significantly. It was found that, for a hydrophilic–hydrophobic contact, water provided a better lubrication effect than oil to reduce the COF.

Pawlak et al. studied the relationship between friction and wettability for the hydrodynamic lubrication regime [90]. Two different aqueous environments were prepared for the tribological tests. Water was used as a lubricant in one aqueous environment, and an aqueous two-phase lubricant (water and additives) was used in the other. A pin-on-disc tribotester was used for the tribological characterization. The tribopair sets (pin and disc) were classified into three groups: hydrophilic–hydrophilic (e.g., steel–steel), hydrophobic–hydrophobic (e.g., PTFE–PTFE), and hydrophobic-hydrophilic (e.g., PTFE–steel) according to their wetting properties. It was found that the delta wettability, Δθ, is the most important wetting parameter to understand the effect of wetting on the COF of sliding surfaces. Delta wettability is the difference of the CA of the two mating surfaces (CA of the tribotester disc—CA of the tribotester pin). For hydrophilic–hydrophilic tribopairs, in the presence of water, the COF is high and increases slightly with Δθ. A thin adhesive layer of water between the two hydrophilic surfaces is responsible for the high COF. For hydrophobic–hydrophobic tribopairs, water cannot stick at the interface. Therefore, the COF does not change notably with increasing Δθ. For hydrophilic–hydrophobic tribopairs, the COF decreases with increasing Δθ. In this case, the adhesive layer of water at the interface is weaker than that of hydrophilic–hydrophilic tribopairs. Therefore, the COF is also lower. Also, the presence of a continuous layer of water at the interface causes liquid slip at the hydrophobic surface. It produces a good lubrication effect, and the COF is reduced.

Kalin and Polajnar found that the wetting properties at the solid–liquid interface affect the friction in oil-lubricated contacts [46]. They measured the COF of steel/steel, steel/diamond-like carbon coating (DLC), and DLC/DLC contacts at an intermediate sliding velocity of 1.2 m/s. For the CA measurements of each combination of oil and selected surface, a CA goniometer was used. The sessile droplet technique was used to determine the surface free energy of the selected surfaces. Using the surface free energy data, the SP was determined. It was observed that, for analyzing the effect of wetting on the COF of sliding surfaces, the SP is more effective than the CA. A small value of the SP corresponds to a low surface free energy. A low surface free energy causes low adhesion between the mating surfaces. As a result, the smaller the value of the SP, the smaller the COF.

Frictional behavior in the presence of lubrication or a cutting fluid is an important topic for different industries. Also, laser texturing technology is becoming popular for synthesizing surfaces with desired optical, mechanical, and chemical properties. Pang et al. studied the effect of wettability on the friction of laser-textured cemented carbide surfaces in dilute cutting fluid [91]. Four different types of cemented carbide samples of different surface textures (e.g., concave and concave) were analyzed. Emulsified oil and water at a volume ratio of 1:40 were used as the cutting fluid. For tribological characterization, the COF of cemented carbide samples were slid against a steel counterface of a pin-on-disc reciprocating tribometer. For characterizing wettability, the CA and delta wettability, Δθ, were measured. For both the CA and the Δθ, an excellent correlation with the COF was observed. For all cemented carbide samples, with a decreasing CA of the cutting fluid with the cemented carbide surface, the COF decreased. The decreasing CA improved the fluid spreading on the surface and helped the creation of a thin fluid film at the contact interface. As a result, the COF was decreased. On the other hand, with a decreasing Δθ, the COF increased. Due to the decrease in Δθ, the absorption of the polar molecules of the cutting fluid on the hydrophilic cemented carbide surface was observed. Consequently, a high viscosity layer was formed at the contact region of the sliding surfaces. The formation of the high viscosity layer increased the COF between the sliding surfaces.

Lanka et al. studied the tribological and wetting properties of TiO_2_-based hydrophobic coatings [71]. To replicate a system for tire–concrete friction in the laboratory setup, nitrile rubber and ceramic tiles were used. For the tribological characterization, the COF between the moving ceramic tile samples and the static rubber pin was measured using a tribometer under dry friction conditions. The wetting was characterized by the CA measured with a goniometer. The roughness parameters of the tile surfaces were quantified from 3D confocal microscopic images.

Different compositions of P25 titanium dioxide (TiO_2_), phosphoric acid (H_3_PO_4_), and water solution were used to synthesize the hydrophilic TiO_2_–phosphate-based coatings for the tile samples. Depending upon water/acid ratio, TiO_2_/acid ratio, and heat treatment duration of the coatings, the hydrophilic samples were named as R2, R5, R7, R9, and O2. To synthesize the hydrophobic coatings, the polymethyl hydrogen siloxane (PMHS) emulsion was applied on the top of the hydrophilic TiO_2_–phosphate layer of the tile samples.

**Figure 7:**
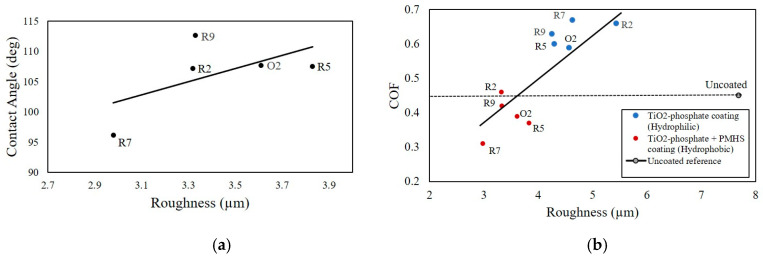
(**a**) Contact angle (CA) vs. surface roughness for hydrophobic tiles and (**b**) COF vs. roughness for hydrophilic and hydrophobic samples (reproduced from Reference [71] with permission).

The effect of surface roughness on the CA of the hydrophobic samples is presented in Figure 7a. From the plot, it is seen that, with the increase in roughness, the CA also increases. Again, an increase in the CA indicates the reduction of the surface free energy. It was found that the surface roughness is inversely related to the surface free energy. However, the relationship between these two parameters can be extremely complex and may depend on different other factors.

The effect of surface roughness on the COF of hydrophilic and hydrophobic samples are presented in Figure 7b. For the hydrophilic coatings, the observed COF values were large and they increased with the increase of surface roughness. TiO_2_ particles sitting on the top of a TiO_2_–phosphate binder of the hydrophilic coatings contributed to the nano- and microroughness. As a result, a notable increase in the COF was observed. For hydrophobic coatings, the COF values were lower compared to the hydrophilic coatings and exhibited an increasing trend with the surface roughness. The thin layer of the PMHS hydrophobic coating smoothens the roughness of the TiO_2_–phosphate layer. In this process, the overall roughness for hydrophobic tile samples was reduced. As a result, the COF values for the hydrophobic samples were decreased.

**Figure 8:**
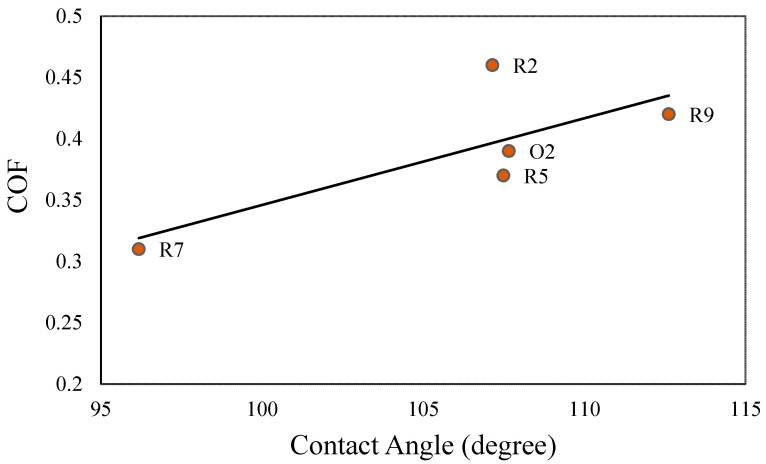
COF vs. CA for hydrophobic tile samples.

To establish a correlation between the friction and wetting of the hydrophobic samples, the COF was plotted against the CA in Figure 8. From the graph, an overall linear relationship between the COF and the CA for the sliding of hydrophobic (TiO_2_–phosphate + PMHS coating) tile samples against a nitrile rubber counterface was observed. With an increase in the CA, the COF values were increased. This relationship of the CA and COF can be explained using the concept of surface roughness. Besides PMHS, the presence of the TiO_2_ nanoparticles on the top of TiO_2_–phosphate binder of the coatings contributed to nano- and microroughness. With increasing nano- and microscale roughness profiles of the hydrophobic tile samples, both the COF and CA were increased.

An important question is whether a hydrophobic coating makes a substrate slippery. The COF of the uncoated tile samples was measured as 0.45. For the hydrophilic samples (TiO_2_–phosphate coated), the measured COF fluctuated between 0.59 to 0.67. For the hydrophobic samples (TiO_2_–phosphate + PMHS coated), the COF fluctuated between 0.31 to 0.46. For the optimal composition of the hydrophobic coating (R2), even a slightly higher COF (0.46) than the uncoated sample was observed. The finding shows that the water-repellent hydrophobic coatings studied in this experiment do not reduce the COF notably and make the surface slippery.

Bhushan and Jung studied the wetting, adhesion, and friction of superhydrophobic and hydrophilic leaves and fabricated micro-/nanopatterned surfaces [92]. They suggested that, during the contact of two hydrophilic bodies, liquid present at the interface forms menisci. The formation of each meniscus depends upon the CA and increases the adhesion and friction.

Sliding of frictional droplets on superhydrophobic surfaces is another important area where interesting observations have been made, for example, that air around droplets rather than the viscosity and pinning can play a major role in frictional dissipation [93] and that droplet friction may have different regimes depending on the viscosity [94] and pinning [95].

From this section, we conclude that numerous attempts have been made to correlate friction with different wetting parameters. However, the scope of the studies was insufficient to find a universal interrelation. The surface roughness and the surface free energy can provide links between friction and wetting; however, the relationship is quite complicated. Extended research is still required to clearly understand the correlations between friction and wetting.

## 6. Conclusions

We discussed how friction is related to wetting, which is important for the development of novel functional surfaces, such as the biomimetic superhydrophobic surfaces. First, we reviewed different basic concepts of friction available in literature. The traditional methods of quantifying friction including the COF, surface free energy, and surface roughness are widely used but have certain limitations. We analyzed the relationship between the surface free energy and friction and discussed mechanisms of friction for different materials. We introduced the basic concepts of wetting and analyzed how the adhesion is related to superhydrophobicity. We also discussed how CAH is related to friction. We reviewed different literature works that correlate friction and wetting. After a detailed study, we concluded the following points.

1. The surface roughness and the surface free energy both contribute to friction and wetting. However, the relationship between these two phenomena may depend on various factors, such as surface chemical heterogeneity, and on various ad hoc parameters.
2. The adhesion and friction between sliding surfaces greatly depends on the surface free energy. A large value of the surface free energy corresponds to strong adhesion, which, in turn, causes high friction between the two surfaces.
3. Surface free energy is not the single parameter that influences the friction between two sliding surfaces. The effect of surface roughness on friction becomes dominant for many materials and tribological systems. With increasing nano- and microscale roughness, both the friction and wetting properties can change.
4. Due to these complex interrelations between different factors, hydrophobic coatings do not necessarily make the surface slippery. For example, if a hydrophobic coating composition incorporates micro- and nanoparticles, surface roughness is induced on the surface. Consequently, the coated surface can be hydrophobic and it can have high friction at the same time.

## Abbreviations

CA	Contact angle
CAH	Contact angle hysteresis
COF	Coefficient of friction
PDMS	Polydimethylsiloxane
PTFE	Polytetrafluoroethylene
PMMA	Polymethylmethacrylate
RMS	Root mean square
AH	Adhesion hysteresis
CL	Contact line
SLIPS	Slippery liquid-infused porous surface
DLC	Diamond-like carbon
SP	Spreading parameter
PMHS	Polymethyl hydrogen siloxane
PC	Polycarbonate
VAMAS	Versailles Project on Advanced Materials and Standards
$R_{t}$	Maximum height of the profile
$R_{sk}$	The skewness parameter
$R_{ku}$	Kurtosis
$R_{q}$	Root mean square roughness
$R_{a}$	Average roughness
$R_{p}$	The highest profile peak
$R_{v}$	The lowest profile valley
$S_{m}$	Mean spacing between peaks
